# Oleic Acid Induces MiR-7 Processing through Remodeling of Pri-MiR-7/Protein Complex

**DOI:** 10.1016/j.jmb.2017.05.001

**Published:** 2017-06-02

**Authors:** Santosh Kumar, Angela Downie Ruiz Velasco, Gracjan Michlewski

**Affiliations:** Wellcome Trust Centre for Cell Biology, University of Edinburgh, Michael Swann Building, Edinburgh, EH9 3BF, UK

**Keywords:** miR, microRNA, RNA-BP, RNA-binding protein, pri-miR, primary miR, pre-miR, precursor miR, GBM, glioblastoma multiforme, HuR, Hu antigen R, MSI2, Musashi homolog2, CTL, conserved terminal loop, OA, oleic acid, RRM, RNA recognition motif, EMSA, electrophoretic mobility shift assay, WEMSA, western blot-combined EMSA, qRT-PCR, quantitative reverse transcription polymerase chain reaction, EA, elaidic acid, miR biogenesis, miR-7, MSI2, HuR, oleic acid (OA)

## Abstract

MicroRNAs (miRs) play a vital role in governing cell function, with their levels tightly controlled at transcriptional and post-transcriptional levels. Different sets of RNA-binding proteins interact with primary miRs (pri-miRs) and precursor-miR transcripts (pre-miRs), controlling their biogenesis post-transcriptionally. The Hu antigen R (HuR)-mediated binding of Musashi homolog2 (MSI2) to the conserved terminal loop of pri-miR-7 regulates the levels of brain-enriched miR-7 formation in a tissue-specific manner. Here, we show that oleic acid (OA) inhibits the binding of proteins containing RNA recognition motifs (RRM) to the conserved terminal loop of pri-miR-7. Using electrophoretic mobility shift assays in HeLa cell extracts, we show that OA treatment disrupts pre-miR/protein complexes. Furthermore, OA rescues *in vitro* processing of pri-miR-7, which is otherwise blocked by HuR and MSI2 proteins. On the contrary, pri-miR-16 shows reduced processing in the presence of OA. This indicates that OA may inhibit the binding of other RRM-containing protein/s necessary for miR-16 processing. Finally, we demonstrate that OA induces mature miR-7 production in HeLa cells. Together, our results demonstrate that OA can regulate the processing of pri-miRs by remodeling their protein complexes. This provides a new tool to study RNA processing and a potential lead for small molecules that target the miR-7 biogenesis pathway.

## Highlights

• OA inhibits the binding of proteins containing RRM.
• OA rescues processing of pri-miR-7 *in vitro*.
• OA induces mature miR-7 production in HeLa cells.

## Graphical Abstract

**Figure:**
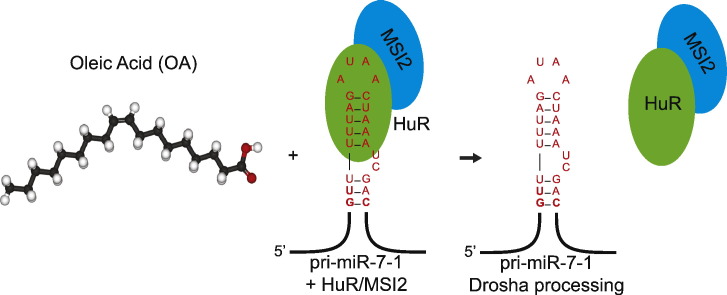

## Introduction

Small non-coding RNAs called microRNAs (miRs) constitute a large family of short (21–23 nt) RNAs that guide gene regulation in diverse and complex patterns [1]. In most metazoan tissues, miRs constitute the dominating class of small RNA regulators [2], [3]. These RNAs regulate the expression of their target mRNAs in complex with ribonucleoproteins also known as miRNPs [4]. MiR-mediated downregulation of gene expression can occur either by decreased mRNA stability or by translational inhibition [5]. Thus, the levels of these small regulators are tightly adjusted to control the final protein levels in the cell. Production of miRs can be controlled either at the transcriptional level by RNA pol II-associated transcription factors or at the post-transcriptional levels to modulate the processing steps of miR biogenesis [2], [6], [7]. Post-transcriptional regulation of miR biogenesis is predominantly achieved by the binding of different classes of RNA-binding proteins (RNA-BPs) to their precursors or primary transcripts and by regulating their stability and subcellular localization [8]. Binding of these RNA-BPs, particularly to the terminal loop of primary miR (pri-miRs) and precursor miRs (pre-miRs), can act as either a repressor or an activator signal for miR biogenesis [9], [10], [11], [12], [13].

Deregulation of microRNA levels is often linked with various pathological conditions including cancer and neurodegenerative disorders [14], [15], [16], [17]. MiR-7, which was originally characterized as a potential tumour suppressor, has been extensively studied in various human cancers and has been shown to regulate diverse biological processes in cancer cells [18], [19]. The biogenesis of miR-7 is regulated at both transcriptional and post-transcriptional levels involving various transcription factors [20], [21], [22] and RNA-BPs [10], [23], [24]. MiR-7 directly binds to the 3′-UTR of the epidermal growth factor receptor mRNA, which leads to the subsequent inhibition of its expression [25]. Notably, inefficient processing of miR-7 has been shown to be the cause of its reduced levels in glioblastoma multiforme (GBM) cells as compared to the surrounding brain tissue [25]. The RNA-BP Hu antigen R (HuR)/ELAV1 has been implicated in regulating miR biogenesis, as knockdown of HuR triggers the upregulation of miR-7 [23]. Recently, we have shown that HuR-mediated recruitment of another RNA-BP, Musashi homolog2 (MSI2), to the conserved terminal loop (CTL) of miR-7 plays an important role in the tissue-specific control of miR-7 biogenesis [10]. Thus, the miR-7–MSI2–HuR ternary complex represents a potential therapeutic target for GBM. HuR has also been implicated in regulating the biogenesis of other miRs, such as miR-133 [26] and miR-199 [27].

Oleic acid (OA), the monounsaturated fatty acid, has recently been shown to inhibit the RNA-binding activity of the MSI1 and MSI2 proteins [28]. It does so by binding to the N-terminal RNA recognition motif (RRM) and inducing an allosteric change to the protein conformation, thereby preventing RNA binding [28]. Thus, in theory, the inhibitory action of OA against the MSI2 protein could be utilized to disrupt miR-7–MSI2–HuR ternary complex formation and rescue the biogenesis of miR-7. On the other hand, elaidic acid (EA), which is a *trans*-isoform of OA and has the same molecular weight, similar refractive index, and molar aqueous solubility as OA, does not bind to MSI1. Fatty acids such as OA and EA occur naturally in various organisms. They are components of membranes, provide energy, and serve as biologically active molecules. Physiologically normal serum contains high μM to low mM concentration of OA [29]. However, during various pathophysiological conditions, the levels of OA are known to rise drastically sometimes up to high mM concentrations [30], [31], [32]. OA makes the highest content of the fatty acids in adipose tissue [33], even though EA is the most abundant *trans*-fatty acid present in the diet [34]. OA is also present in intracellular lipid droplets [35].

Here, using electrophoretic mobility shift assay (EMSA) analysis we show that both OA and EA can inhibit the binding of RNA-BPs to pre-miR-7. Moreover, *in vitro* processing of pri-miR-7 in HeLa cell extracts demonstrates that the presence of OA rescues miR-7 maturation. We show that the treatment of HeLa cells with OA but not EA induces the production of miR-7. Finally, we show that OA does not affect the levels of several control miRs. These results suggest that OA can be important and a specific regulator of RNA processing events.

## Results and discussion

### OA (18:1 ω-9) monounsaturated fatty acid potently inhibits the binding of RNA-BPs to pre-miR-7-1

**Fig. 1:**
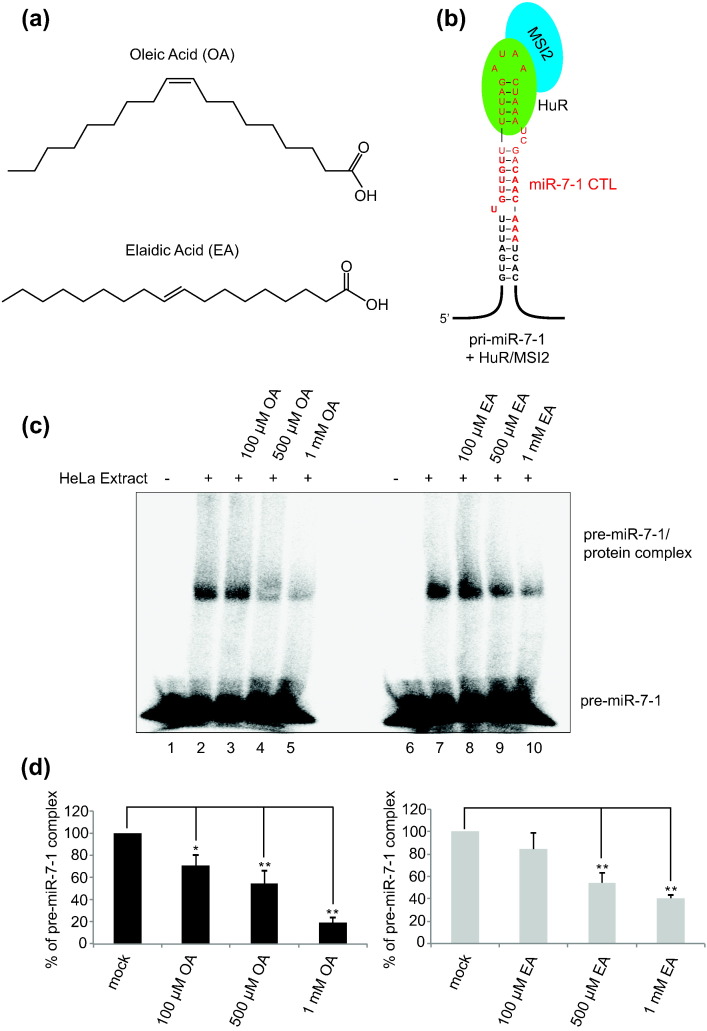
Oleic acid and elaidic acid remodel the pri-miR-7-1/protein complex (a) Molecular structure of OA and EA. (b) Schematic of the secondary structure of pri-miR-7 and CTL of miR-7 (in red) showing the experimentally derived model for pre-miR-7-1/HuR/MSI2 complex (Choudhury *et al.* 2013) [10]. (c) Electrophoretic mobility shift assay (EMSA) of pre-miR-7-1 in the presence of HeLa cell extracts to demonstrate the binding of proteins and to quantitate the effect of increasing concentrations of OA and EA treatment on the pre-miR-7-1/protein complex. The image is a representative of three independent experiments. Lanes 1 and 6 show EMSA with no extract. Lanes 2 and 7 show EMSA with HeLa extract only. Lanes 3, 4, and 5 represent EMSA with increasing concentrations of OA. Lanes 8, 9, and 10 represent EMSA with increasing concentrations of EA. (d) Densitometry analysis of EMSA results to quantitate the effect of OA and EA treatment. Mock control represents the pre-miR-7-1 incubated with HeLa cell extract only. OA and EA treatment values were normalized to values derived from mock treatment. The standard error of the mean (± SEM) was calculated using the replicates of three independent experiments. Student's *t*-test was performed to assess the statistical significance; *P < 0.05, **P < 0.01.

OA is the most abundant and widely distributed fatty acid in nature. It is an 18-carbon monounsaturated fatty acid with one double bond present at the ninth carbon atom from the aliphatic omega end of the molecule (18:1, ω-9) (Fig. 1a). It has been shown to inhibit the binding of MSI1 and MSI2 proteins to their mRNA target in a very strong and specific manner [28]. On the other hand, EA, which is a ω-9 *trans* isomer of OA, demonstrated no inhibition at all. Our previous results showed that pri-miR-7-1 and miR-7-1 CTL bind HuR and MSI2 proteins (Fig. 1b) [10]. To test if fatty acids interfere with pri-miR-7/protein complex, we performed EMSA experiments to monitor the binding of cognate proteins to the pre-miR-7. Incubation of pre-miR-7 with HeLa cell extract shifted the free RNA, indicating the binding of specific proteins to the RNA structure (Fig. 1c). Treatment with OA showed a decrease in the intensity of the shifted band in a concentration-dependent manner (Fig. 1d), suggesting the inhibition of complex formation between proteins and precursor miR-7. This observation supports the earlier findings by Clingman *et al*. where they showed that OA binds to MSI2 and induces an allosteric conformational change to inhibit RNA binding. However, in contrast to the earlier report, EA treatment also inhibited the binding of cognate proteins in a concentration-dependent manner (Fig. 1c). Densitometry analysis showed that the effect of the EA treatment is relatively smaller compared to OA at the same concentrations (Fig. 1d).

**Fig. 2:**
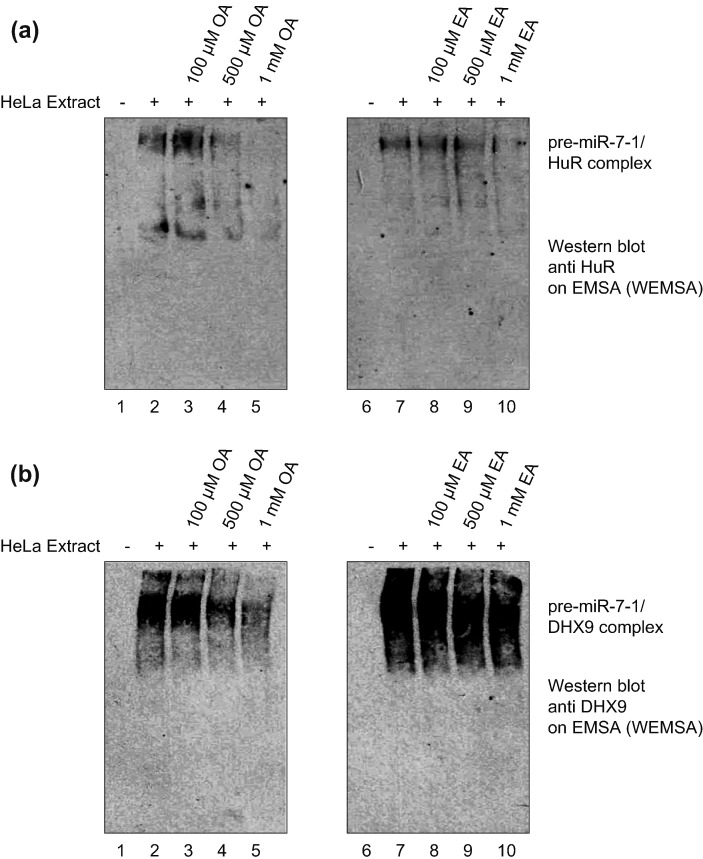
HuR and DHX9 protein are detected in the pre-miR-7/protein complex. The figure shows western blot-combined EMSA (WEMSA) of EMSA presented in Fig. 1c. (a) HuR and (b) DHX9 were detected using corresponding antibodies. Lanes 1 and 6 show WEMSA with no extract. Lanes 2 and 7 show WEMSA with HeLa extract only. Lanes 3, 4, and 5 represent WEMSA with increasing concentrations of OA. Lanes 8, 9, and 10 represent WEMSA with increasing concentrations of EA. All WEMSA blots were repeated for a minimum of three times.

**Fig. 3:**
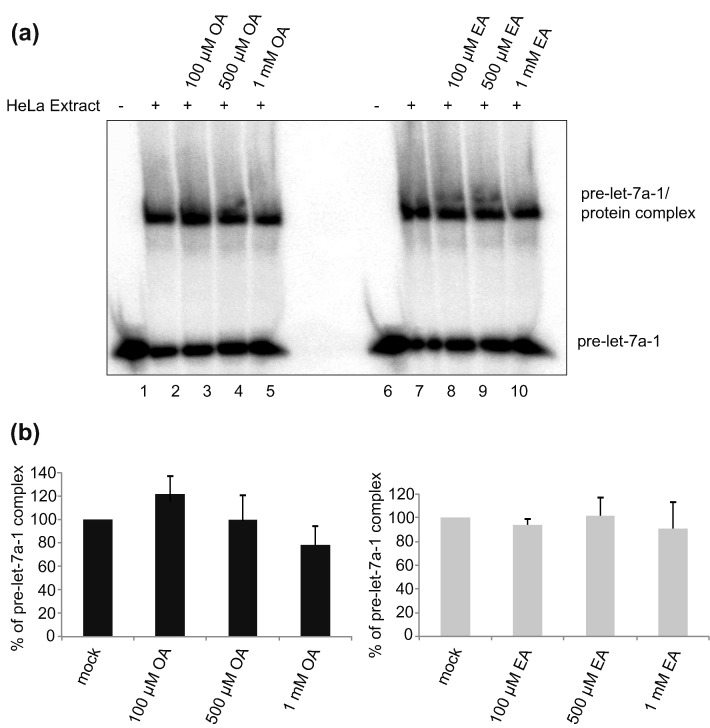
Oleic acid and elaidic acid do not remodel the pre-let-7a-1/protein complex. (a) Electrophoretic mobility shift assay (EMSA) of pre-let-7a-1 in the presence of HeLa cell extracts and increasing concentrations of OA and EA treatment. The image is a representative of three independent experiments. Lanes 1 and 6 show EMSA with no extract. Lanes 2 and 7 show EMSA with HeLa extract only. Lanes 3, 4, and 5 represent EMSA with increasing concentrations of OA. Lanes 8, 9, and 10 represent EMSA with increasing concentrations of EA. (b) Densitometry analysis of EMSA results to quantitate the effect of OA and EA treatment. Mock control represents the pre-let-7a-1 incubated with HeLa cell extract only. OA and EA treatment values were normalized to values derived from mock treatment. The standard error of the mean (± SEM) was calculated using the replicates of three independent experiments.

**Fig. 4:**
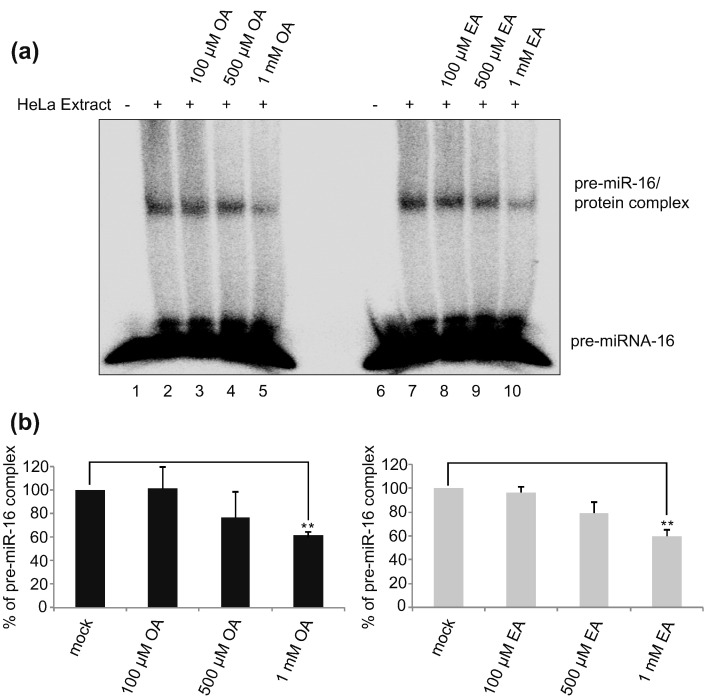
High concentrations of oleic acid (OA) and elaidic acid (EA) affect the pre-miR-16/protein complex. (a) Electrophoretic mobility shift assay (EMSA) of pre-miR-16 in the presence of HeLa cell extracts and increasing concentrations of OA and EA treatment. The image is a representative of three independent experiments. Lanes 1 and 6 show EMSA with no extract. Lanes 2 and 7 show EMSA with HeLa extract only. Lanes 3, 4, and 5 represent EMSA with increasing concentrations of OA. Lanes 8, 9, and 10 represent EMSA with increasing concentrations of EA. (b) Densitometry analysis of EMSA results to quantitate the effect of OA and EA treatment. Mock control represents the pre-miR-16 incubated with HeLa cell extract only. OA and EA treatment values were normalized to values derived from mock treatment. The standard error of the mean (± SEM) was calculated using the replicates of three independent experiments. Student's *t*-test was performed to assess the statistical significance; **P < 0.01.

Next to validate that the aggregates seen in EMSA are RNA-BPs interacting with pre-miR-7, we used a modified EMSA with pre-miR-7, which was transferred to nitrocellulose and blotted against HuR and DHX9 (Fig. 2). This approach, called western blot-combined EMSA (WEMSA), positively identified HuR (Fig. 2a) and DHX9 (Fig. 2b) as components of the pre-miR-7/protein complex in HeLa cell extracts. Furthermore, it also revealed that OA treatment inhibited HuR binding to pre-miRs to much greater extent than EA. Notably, control EMSA with pre-let-7a-1 and pre-miR-16 showed that both OA and EA treatments did not significantly affect the RNA–protein complexes formed by pre-let-7a-1 (Fig. 3) and affected the pre-miR-16 complex only at the 1 mM concentration (Fig. 4). These results indicate that there is some level of selectivity of OA and EA toward pre-miR-7 *in vitro*.

The fact that both OA and EA are disrupting pre-miR-7/protein complex disagrees with the earlier observation by Clingman and colleagues [28], where they observed no inhibition of RNA–protein interaction upon EA treatment, which could arise from experimental factors. One explanation for the observed differences in EA action could be the fact that they worked with purified MSI2 protein, whereas we monitored the binding of protein factors extracted from HeLa cells. Thus, the difference in the inhibitory potency of the two fatty acids could possibly stem from their differential binding strength to endogenous proteins as compared to recombinant proteins. Moreover, we used much higher concentrations of OA and EA to observe the effects. In the present study, the solubility of these fatty acids was not measured, but earlier report suggests that OA and EA both have similar molar aqueous solubility (~ 10 mM at pH 8.0) [28]. Thus, our working concentration of OA and EA was well below the maximum aqueous solubility of these acids, which dismisses the possibility of insoluble aggregate formation.

### OA disrupts the binding of RRM-containing proteins to the CTL of pri-miR-7-1

**Fig. 5:**
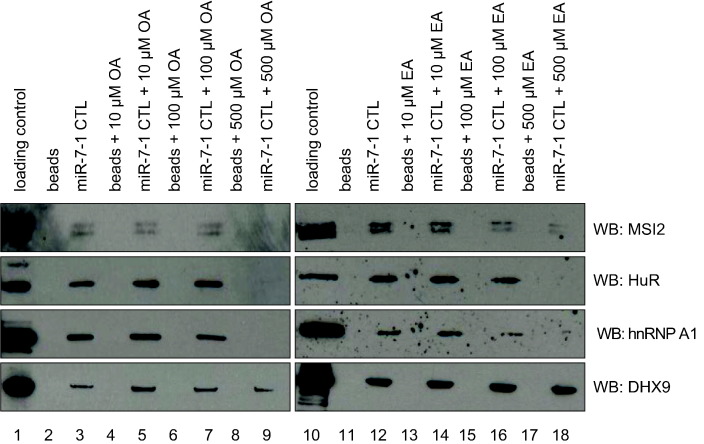
OA and EA inhibit the binding of RRM contacting proteins to the miR-7-1 CTL. The figure shows western blot analysis of miR-7-1 CTL RNA pull-down with HeLa cell extract and OA/EA treatment. Selected proteins (MSI2, HuR, hnRNPA1, and DHX9) were monitored using western blot analysis with corresponding antibodies. Lanes 1 and 10 represent the loading control of HeLa cell extract; Lanes 2 and 11 show beads alone. Lanes 4 and 13, 6 and 15, and 8 and 17 show beads incubated with 10 μM, 100 μM, and 500 μM of OA and EA, respectively. Lanes 3 and 12 represent miR-7-1 CTL pull-down. Lanes 5 and 14, 7 and 16, and 9 and 18 represent the miR-7-1 CTL pull-down in HeLa cell extract treated with 10 μM, 100 μM, and 500 μM of OA and EA, respectively. The experiment has been repeated three times.

Next, to investigate the effects of OA/EA on pre-miR-7–protein complex, we performed RNA pull-down assay in HeLa cell extracts using the miR-7 CTL conjugated to agarose beads. This assay allows for efficient and specific capture of the proteins bound to the candidate RNA molecule. Western blot analysis was performed for selected RRM-containing RNA-BPs (MSI2, HuR, and hnRNP A1) and a double-stranded RNA-specific binding protein DHX9, which is a DEAH-box-containing RNA helicase. These proteins were chosen based on an earlier report where they were shown to interact specifically with the miR-7 CTL [10]. Another reason for choosing these proteins is that OA is known to inhibit the binding of MSI1 and MSI2 by binding to their RRMs, thereby inducing a conformational change to prevent RNA binding [28]. HuR, MSI2, and hnRNP A1 proteins showed no significant effect in their RNA pull-down assay at lower concentrations of OA and EA treatment (Fig. 5). However, with 500 μM of OA, the binding of HuR, MSI2, and hnRNP A1 proteins was completely abolished (Fig. 5). EA treatment at 500 μM also resulted in the complete abolishment of HuR and hnRNP A1 binding to the miR-7 CTL, while MSI2 binding was reduced. Additionally, the binding of DHX9 was monitored to examine if OA and EA treatment inhibit the binding of other types of RNA-BPs or only the RRM-containing proteins. Western blot analysis of DHX9 showed no effect of OA and EA treatment on DHX9 binding to the CTL of miR-7 (Fig. 5). This suggests that differential binding of OA and EA to RRM-containing proteins leads to the inhibition of RNA–protein complex formation; however, double-stranded RNA-BP remains unaffected. This also explains the disparity in concentrations of the fatty acids needed to achieve the complete dissociation of all proteins in EMSA assays. In the EMSA experiment, there was a gradual reduction in the total amount of proteins bound to the RNA structure even at 1 mM of acid treatment, suggesting that the fatty acid treatment does not inhibit the binding of all the RNA-BPs.

### The biogenesis of miRs in HeLa cell extract is influenced by OA and EA treatment

**Fig. 6:**
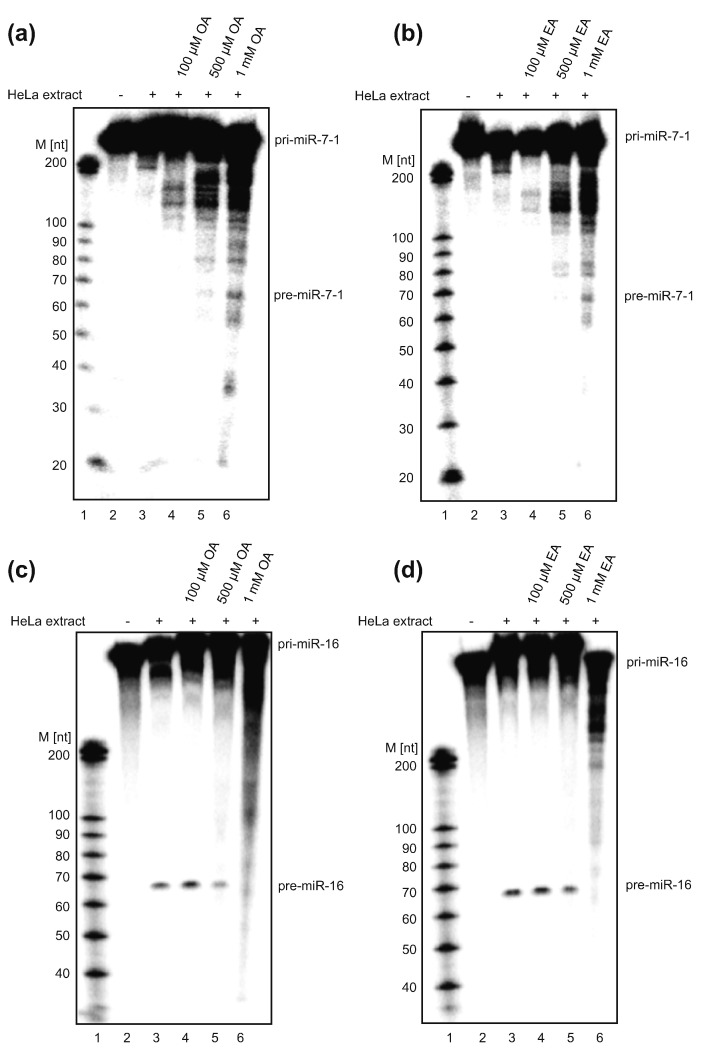
OA induces pri-miR-7-1 processing *in vitro*. (a and b) *In vitro* processing of pri-miR-7-1 and (c and d) pri-miR-16 with HeLa extract in the presence of different concentrations of OA and EA. The processing of pri-miR-7-1 is increased upon (a) OA and (b) EA treatment. Radiolabeled pri-miR-7-1 (~ 30 × 10^3^ cpm) transcript was incubated with 50% (wt/vol) total HeLa cell extract, and the concentration-dependent effect of (a) OA and (b) EA treatment was assessed on the processing of pri-miR-7-1. The *in vitro* processing of pri-miR-16 is inhibited upon (c) OA and (d) EA treatment. Radiolabeled pri-miR-16 (~ 30 × 10^3^ cpm) was incubated with the 50% (wt/vol) total HeLa cell extract, and the concentration-dependent effect of (a) OA and (b) EA treatment was assessed on the processing of pri-miR-16. All the products of *in vitro* transcription were resolved on 8% denaturing polyacrylamide gel. Lane 1 in each gel represents the decade marker for RNA size. Lanes 2 and 3 in each gel represent mock treatment and *in vitro* processing reactions, respectively. Lanes 4 and 5 show *in vitro* processing reaction in increasing concentrations of OA and EA as indicated in the figure. These experiments were repeated for a minimum of three times.

As evident from earlier studies, the biogenesis of miR-7 is altered in HeLa cells, owing to the presence of MSI2 and HuR bound to the CTL of the pri-miR-7 [10]. Binding of these *trans*-acting proteins inhibits the microprocessor-mediated processing. Thus, OA and EA could be used to restore miR-7 biogenesis. To verify this, we performed *in vitro* processing of pri-miR-7-1 in HeLa cell extracts in the presence of increasing concentrations of OA and EA. We showed that treatment with both OA and EA could rescue miR-7 processing (Fig. 6a and b). The effect was only seen at 1 mM fatty acid concentration most likely arising from reduced sensitivity of *in vitro* processing assays. Binding of *trans*-acting proteins to the terminal loop of pri-miR-7-1 was inhibited by OA and EA, thus leading to a noticeable increase in microprocessor activity generating pre-miR-7. Importantly, these results demonstrate that the presence of OA and EA does not inhibit Drosha. We speculate that OA (and likely EA also) binds to RRM-containing RNA-BPs only and does not affect the binding of other RNA-BPs that are necessary for the microprocessor activity [36].

### The biogenesis of miR-16 is inhibited by OA and EA treatment

To obtain more mechanistic insights into OA and EA binding, we chose to analyze the processing of miR-16. The levels of miR-16 remained unaffected upon MSI2 or HuR downregulation [10]. Moreover, miR-16 has been used in many studies as an internal control, as it is ubiquitously expressed and its level remains relatively stable during the cell life cycle [37], [38]. Interestingly, when we performed *in vitro* processing of pri-miR-16 in the presence of OA and EA, we observed concentration-dependent inhibition of pri-miR-16 processing (Fig. 6c and d). This result implies that RRM-containing protein factor/s could regulate the biogenesis of miR-16. However, further experiments, which are needed to test this hypothesis, are beyond the scope of this paper. Nonetheless, these results clearly demonstrate that different sets of *trans*-acting proteins are required to regulate biogenesis of various miRs.

### OA treatment rescues mature miR-7 production in cells

**Fig. 7:**
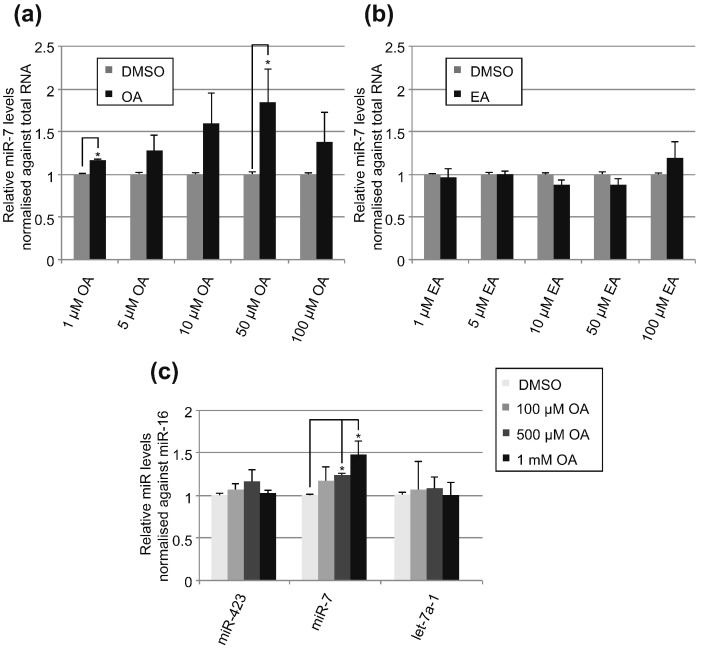
OA induces miR-7 production in HeLa cells. Real-time qRT-PCR of miR-7 upon treatment of HeLa cells with (a) OA and (b) EA for 24 h. Mock controls were treated with the same volume of pure DMSO as OA/EA treatments. The change in levels of miR-7 was calculated relative to the mock treatment. The level was normalized to total RNA. Error bars represent the standard error of the mean (± SEM) of three independent experimental repeats. Statistical analysis was performed by student's *t*-test *P < 0.05. (c) Real-time qRT-PCR of selected miRs upon OA treatment of HeLa cells culture in Opti-MEM without serum. The change in levels of miRs was calculated relative to the DMSO treatment. The level was normalized to miR-16. Error bars represent the standard error of the mean (± SEM) of three independent experimental repeats. Statistical analysis was performed by student's *t*-test *P < 0.05.

The effect of OA and EA treatment was assessed in HeLa cells by monitoring the levels of mature miR-7 using quantitative reverse transcription polymerase chain reaction (qRT-PCR). Previously, it was shown that downregulation of MSI2 or HuR individually or in combination resulted in the rescue of pri-miR-7 processing, leading to a twofold increase in the levels of mature miR-7 [10]. We subsequently treated HeLa cells with various concentrations of OA and EA for 24 h and monitored the levels of mature miR-7 by qRT-PCR. At concentrations higher than 100 μM, the cells were not viable; thus, in standard cell culture conditions, we had to use suboptimal concentrations of OA and EA. Additionally, we chose to normalize the miR-7 levels to total RNA to avoid the possibility of bias resulting from OA-mediated regulation of other endogenous RNAs. Importantly, these results showed a concentration-dependent increase in the levels of miR-7 at lower concentrations of OA, up to 50 μM (Fig. 7a). However, 100 μM OA treatment exhibited a smaller increase in miR-7 levels, suggesting additional and toxic effects of OA at this concentration (Fig. 7a). Interestingly, EA did not trigger an increase in the miR-7 levels in concentrations up to 100 μM (Fig. 7b). Notably, standard cell culture medium already contains high levels of fatty acids [29]. For this reason, we modified our analysis in HeLa cells and cultured them in Opti-MEM without serum. This allowed us to use similar OA concentrations to those from the *in vitro* experiments, without compromising cellular fitness. This resulted in a smaller but still statistically significant increase of mature miR-7 upon OA treatment (Fig. 7c). In these conditions, miR-16 levels did not change, and thus, we have used it as a normalization control toward let-7a-1 and miR-423, which also remained constant (Fig. 7c). Together, these results clearly demonstrate that OA treatment increases the levels of mature miR-7 in HeLa cells. Moreover, this also indicates that the presence of OA does not alter the global activity of the microprocessor. This is important as microprocessor has been shown to be involved other cellular processes such as splicing [39] or gene expression [40].

The levels of polyunsaturated fatty acids, for which OA is a precursor, are more tightly controlled than saturated fatty acids, owing to their diverse roles in the mammalian physiology. OA treatment on the glial cells suppresses lipogenesis and cholesterologensis [41]. In colorectal cancer patients, the levels of OA are significantly higher than the levels of most saturated fatty acids [42]. On the contrary, OA levels are significantly reduced in Alzheimer's disease patients [43]. Thus, it is possible that fluctuation of the OA levels can change cellular metabolism, which could lead to pathophysiological conditions. Studying the effects of fatty acids, such as OA, on the miR biogenesis and RNA processing in general could uncover novel regulatory pathways involved in cellular homeostasis. These studies could also provide an entry point toward designing potential drug molecules that target miR biogenesis pathway. In the case of GBM, the levels of miR-7 are post-transcriptionally downregulated [25]. Therefore, drugs based on natural compounds, such as OA, could bypass delivery issues surrounding miR replacement strategies in the brain. This would be feasible as fatty acids are known to cross the blood brain barrier [44]. In summary, our results suggest that monounsaturated fatty acids can regulate the binding of RRM-containing RNA-BPs to their target RNAs and that any change in the cellular concentration of fatty acids might lead to complex rewiring of the cellular processes. Further work needs to be done to understand the specific roles of fatty acids in regulating RNA processing.

In this study, we have explored the inhibitory action of OA against the RNA-BPs involved in the inhibition of miR-7 biogenesis. We demonstrated that OA not only inhibits the binding of MSI2 protein but also interferes with other RRM-containing RNA-BPs known to bind to the miR-7 CTL. More interestingly, OA treatment showed opposite effects on the *in vitro* processing of the pri-miR-7 and pri-miR-16. This indicates various requirements for the *trans*-acting protein factors that play positive and negative roles in the control of their processing. Further studies need to be performed to unravel the specific proteins necessary for the regulation of miR-16 biogenesis. Earlier [28], EA had been shown to have no effect on the MSI2 RNA-binding activity. However, we found that EA also inhibits the binding of RRM-containing RNA-BPs, although in higher concentrations and to a lower extent. We propose that OA can be used as a molecular tool to study the regulation of RNA processing and to provide an entry point into designing therapies targeted at miR-7 biogenesis pathways.

## Materials and methods

### Primers and RNA sequences

The CTL sequence of miR-7-1 (5′-UGU UGU UUU UAG AUA ACU AAA UCG ACA ACA AA- 3′) was purchased from Sigma Aldrich and dissolved in water to make a 1 nmol/μL stock. The pri-miR-7-1, pre-miR-7-1, pre-miR-16, and pre-let-7a-1 sequences were amplified from the pCG-T7 mammalian expression vectors containing corresponding sequences and prepared as described previously [10].

### RNA pull-down assay and western blot analysis

RNA pull-down assays were based on a method described previously [45]. HeLa cell extracts were prepared by collecting ~ 3 × 10^6^ cells in 1 mL of buffer D (20 mM Tris–HCl at pH 7.9, 20% p/v glycerol, 0.1 M KCl, 0.2 mM EDTA, 0.5 mM DTT, and 0.2 mM PMSF) and sonicating 10 × 30 s on/off using a temperature-controlled water bath sonicator. Immobilization of RNA to agarose beads and affinity purification of proteins was performed using a previously published protocol [46]. OA [≥ 99% (GC)] and EA [≥ 99% (GC)] were purchased from Sigma-Aldrich. Following the pull-down experiment, the proteins were separated using a 4–12% gradient Bis-tris gel (NuPAGE Novex, Invitrogen) and transferred on to a nitrocellulose membrane (Whatman) in 25 mM Tris-base, 40 mM glycine, and 10% (vol/vol) methanol using a Genie blotter unit (Idea scientific company) at 12 V for 1 h. After transfer, the membrane was blocked with western blocking solution (Roche) diluted in 1:10 using TBS-T buffer [20 mM Tris at pH 7.5, 137 mM NaCl, and 0.1% (vol/vol) Tween 20]. Specific proteins were detected using the primary antibodies in western blocking solution (diluted 1:20 in TBS-T) and incubated for 1 h at room temperature. The following dilutions of the primary antibodies were used: rabbit polyclonal anti-MSI2 (1:1000; clone EP1305Y, Millipore), rabbit polyclonal anti-HuR (1:1000; Millipore), rabbit polyclonal anti-DNX9 (1:1000; Proteintech), and mouse monoclonal anti-hnRNP A1 (clone 4B10; 1:1000; Santa Cruz Biotechnology). After incubation in primary antibodies, the blot was washed with TBS-T for 3 × 5 min and incubated with the appropriate secondary antibody conjugated with horseradish peroxidase. These bound secondary antibodies were then visualized using SuperSignal west Pico detection reagent (Thermo Scientific) and film. The membrane was reblotted after stripping with ReBlot Plus Strong antibody-stripping solution (Chemicon) diluted 1:10 in water and by blocking the membrane using western blocking solution in TBS-T.

### EMSA and WEMSA

Pre-miRs were synthesized by *in vitro* transcription, and ^32^P α-UTP was used for the body labeling of the RNA. Radiolabeled pre-miRs were purified by running 10% denaturing PAGE in 1 × TBE buffer (89 mM Tris at pH 7.5, 89 mM boric acid, and 2 mM EDTA). Radiolabeled pre-miRs were incubated with HeLa cell extract in the absence and presence of different concentrations of the OA and EA on ice for 1 h before loading onto the gel. Free and protein bound RNA was separated on 6% non-denaturing PAGE by running in 0.5 × TBE at 8 W. The gel-exposed phosphor screen was scanned using a Fujifilm FLA 5000 scanner. Image analysis and quantitation were performed using Aida Image analyser V.4.27 software. For WEMSA, after PAGE, the proteins were transferred onto a nitrocellulose membrane. Subsequent protein detection was carried out as in western blot analysis.

### *In vitro* processing assay

Pri-miR-7-1 and pri-miR-16 were synthesized by *in vitro* transcription using plasmids containing respective sequences as templates in the presence of ^32^P α-UTP [10]. Radiolabeled pri-miRs (~ 30,000 c.p.m.) were incubated with 50% HeLa cell extract in either the presence or absence of OA and EA, and processing was performed at 37 °C for 30 min with 0.5 mM ATP, 20 mM creatine phosphate, and 3.2 mM MgCl_2_. Next, phenol chloroform extraction was performed, followed by precipitation and separation on 8% denaturing PAGE in 1 × TBE.

### Real-time qRT-PCR

HeLa cells were seeded in a 6-well plate and treated with different concentrations of OA/EA (dissolved in pure DMSO) at ~ 80% confluency. After 24 h, total RNA was extracted using TRIzol reagent (Invitrogen) following the manufacturer's instructions. The concentration of total RNA was measured using a NanoDrop 1000 spectrophotometer. Equal amounts (400 ng) of total RNA were used for the cDNA synthesis using the miScript Reverse Transcription Kit according to the manufacturer's instructions (Qiagen). Real-time PCR was performed using 2 × QuantiTect SYBR Green PCR Master Mix, miScript universal primer, and primers for corresponding miRs. We could not assume that OA treatment is neutral toward selected small RNAs. For this, the levels of miR-7 were normalized to the amount of total RNA loaded to each reaction. Alternatively, we performed similar analyses in HeLa cells grown in Opti-MEM without serum. Here, we used miR-16 as a loading control.

## References

[1] Ghildiyal M., Zamore P.D. (2009). Small silencing RNAs: an expanding universe. Nat. Rev. Genet..

[2] Ha M., Kim V.N. (2014). Regulation of microRNA biogenesis. Nat. Rev. Mol. Cell Biol..

[3] Pasquinelli A.E. (2015). MicroRNAs: heralds of the noncoding RNA revolution. RNA.

[4] Steitz J.A., Vasudevan S. (2009). miRNPs: versatile regulators of gene expression in vertebrate cells. Biochem. Soc. Trans..

[5] Valinezhad Orang A., Safaralizadeh R., Kazemzadeh-Bavili M. (2014). Mechanisms of miR-mediated gene regulation from common downregulation to mRNA-specific upregulation. Int. J. Genomics.

[6] Krol J., Loedige I., Filipowicz W. (2010). The widespread regulation of microRNA biogenesis, function and decay. Nat. Rev. Genet..

[7] Finnegan E.F., Pasquinelli A.E. (2013). MicroRNA biogenesis: regulating the regulators. Crit. Rev. Biochem. Mol. Biol..

[8] Siomi H., Siomi M.C. (2010). Posttranscriptional regulation of microRNA biogenesis in animals. Mol. Cell.

[9] Castilla-Llorente V., Nicastro G., Ramos A. (2013). Terminal loop-mediated regulation of miR biogenesis: selectivity and mechanisms. Biochem. Soc. Trans..

[10] Choudhury N.R., de Lima Alves F., de Andres-Aguayo L., Graf T., Caceres J.F., Rappsilber J. (2013). Tissue-specific control of brain-enriched miR-7 biogenesis. Genes Dev..

[11] Guil S., Caceres J.F. (2007). The multifunctional RNA-binding protein hnRNP A1 is required for processing of miR-18a. Nat. Struct. Mol. Biol..

[12] Lunse C.E., Michlewski G., Hopp C.S., Rentmeister A., Caceres J.F., Famulok M. (2010). An aptamer targeting the apical-loop domain modulates pri-miR processing. Angew. Chem..

[13] Michlewski G., Guil S., Semple C.A., Caceres J.F. (2008). Posttranscriptional regulation of miRs harboring conserved terminal loops. Mol. Cell.

[14] Hata A., Lieberman J. (2015). Dysregulation of microRNA biogenesis and gene silencing in cancer. Sci. Signal..

[15] Abe M., Bonini N.M. (2013). MicroRNAs and neurodegeneration: role and impact. Trends Cell Biol..

[16] Gommans W.M., Berezikov E. (2012). Controlling miR regulation in disease. Methods Mol. Biol..

[17] Castello A., Fischer B., Hentze M.W., Preiss T. (2013). RNA-binding proteins in Mendelian disease. Trends Genet..

[18] Gu D.N., Huang Q., Tian L. (2015). The molecular mechanisms and therapeutic potential of microRNA-7 in cancer. Expert Opin. Ther. Targets.

[19] Horsham J.L., Kalinowski F.C., Epis M.R., Ganda C., Brown R.A., Leedman P.J. (2015). Clinical potential of microRNA-7 in cancer. J. Clin. Med..

[20] Reddy S.D., Ohshiro K., Rayala S.K., Kumar R. (2008). MicroRNA-7, a homeobox D10 target, inhibits p21-activated kinase 1 and regulates its functions. Cancer Res..

[21] Chou Y.T., Lin H.H., Lien Y.C., Wang Y.H., Hong C.F., Kao Y.R. (2010). EGFR promotes lung tumorigenesis by activating miR-7 through a Ras/ERK/Myc pathway that targets the Ets2 transcriptional repressor ERF. Cancer Res..

[22] McInnes N., Sadlon T.J., Brown C.Y., Pederson S., Beyer M., Schultze J.L. (2012). FOXP3 and FOXP3-regulated microRNAs suppress SATB1 in breast cancer cells. Oncogene.

[23] Lebedeva S., Jens M., Theil K., Schwanhausser B., Selbach M., Landthaler M. (2011). Transcriptome-wide analysis of regulatory interactions of the RNA-binding protein HuR. Mol. Cell.

[24] Wang Y., Vogel G., Yu Z., Richard S. (2013). The QKI-5 and QKI-6 RNA binding proteins regulate the expression of microRNA 7 in glial cells. Mol. Cell. Biol..

[25] Kefas B., Godlewski J., Comeau L., Li Y., Abounader R., Hawkinson M. (2008). microRNA-7 inhibits the epidermal growth factor receptor and the Akt pathway and is down-regulated in glioblastoma. Cancer Res..

[26] Legnini I., Morlando M., Mangiavacchi A., Fatica A., Bozzoni I. (2014). A feedforward regulatory loop between HuR and the long noncoding RNA linc-MD1 controls early phases of myogenesis. Mol. Cell.

[27] Zhang L.F., Lou J.T., Lu M.H., Gao C., Zhao S., Li B. (2015). Suppression of miR-199a maturation by HuR is crucial for hypoxia-induced glycolytic switch in hepatocellular carcinoma. EMBO J..

[28] Clingman C.C., Deveau L.M., Hay S.A., Genga R.M., Shandilya S.M., Massi F. (2014). Allosteric inhibition of a stem cell RNA-binding protein by an intermediary metabolite. elife.

[29] Abdelmagid S.A., Clarke S.E., Nielsen D.E., Badawi A., El-Sohemy A., Mutch D.M. (2015). Comprehensive profiling of plasma fatty acid concentrations in young healthy Canadian adults. PLoS One.

[30] Fagot-Campagna A., Balkau B., Simon D., Warnet J.M., Claude J.R., Ducimetiere P. (1998). High free fatty acid concentration: an independent risk factor for hypertension in the Paris prospective study. Int. J. Epidemiol..

[31] Artwohl M., Roden M., Waldhausl W., Freudenthaler A., Baumgartner-Parzer S.M. (2004). Free fatty acids trigger apoptosis and inhibit cell cycle progression in human vascular endothelial cells. FASEB J..

[32] Reaven G.M., Hollenbeck C., Jeng C.Y., Wu M.S., Chen Y.D. (1988). Measurement of plasma glucose, free fatty acid, lactate, and insulin for 24 h in patients with NIDDM. Diabetes.

[33] Kokatnur M.G., Oalmann M.C., Johnson W.D., Malcom G.T., Strong J.P. (1979). Fatty acid composition of human adipose tissue from two anatomical sites in a biracial community. Am. J. Clin. Nutr..

[34] Tardy A.L., Morio B., Chardigny J.M., Malpuech-Brugere C. (2011). Ruminant and industrial sources of trans-fat and cardiovascular and diabetic diseases. Nutr. Res. Rev..

[35] Rohwedder A., Zhang Q., Rudge S.A., Wakelam M.J. (2014). Lipid droplet formation in response to oleic acid in Huh-7 cells is mediated by the fatty acid receptor FFAR4. J. Cell Sci..

[36] Gregory R.I., Yan K.P., Amuthan G., Chendrimada T., Doratotaj B., Cooch N. (2004). The microprocessor complex mediates the genesis of microRNAs. Nature.

[37] McDonald J.S., Milosevic D., Reddi H.V., Grebe S.K., Algeciras-Schimnich A. (2011). Analysis of circulating microRNA: preanalytical and analytical challenges. Clin. Chem..

[38] Peltier H.J., Latham G.J. (2008). Normalization of microRNA expression levels in quantitative RT-PCR assays: identification of suitable reference RNA targets in normal and cancerous human solid tissues. RNA.

[39] Havens M.A., Reich A.A., Hastings M.L. (2014). Drosha promotes splicing of a pre-microRNA-like alternative exon. PLoS Genet..

[40] Gromak N., Dienstbier M., Macias S., Plass M., Eyras E., Caceres J.F. (2013). Drosha regulates gene expression independently of RNA cleavage function. Cell Rep..

[41] Natali F., Siculella L., Salvati S., Gnoni G.V. (2007). Oleic acid is a potent inhibitor of fatty acid and cholesterol synthesis in C6 glioma cells. J. Lipid Res..

[42] Baro L., Hermoso J.C., Nunez M.C., Jimenez-Rios J.A., Gil A. (1998). Abnormalities in plasma and red blood cell fatty acid profiles of patients with colorectal cancer. Br. J. Cancer.

[43] Fonteh A.N., Cipolla M., Chiang J., Arakaki X., Harrington M.G. (2014). Human cerebrospinal fluid fatty acid levels differ between supernatant fluid and brain-derived nanoparticle fractions, and are altered in Alzheimer's disease. PLoS One.

[44] Guest J., Garg M., Bilgin A., Grant R. (2013). Relationship between central and peripheral fatty acids in humans. Lipids Health Dis..

[45] Michlewski G., Cáceres J.F. (2010). RNase-assisted RNA chromatography. RNA.

[46] Choudhury N.R., Nowak J.S., Zuo J., Rappsilber J., Spoel S.H., Michlewski G. (2014). Trim25 is an RNA-specific activator of Lin28a/TuT4-mediated uridylation. Cell Rep..

